# Experiences and perceptions about cause and prevention of cardiovascular disease among people with cardiometabolic conditions: findings of in-depth interviews from a peri-urban Nepalese community

**DOI:** 10.3402/gha.v7.24023

**Published:** 2014-04-30

**Authors:** Natalia Oli, Abhinav Vaidya, Madhusudan Subedi, Alexandra Krettek

**Affiliations:** 1 Department of Community Medicine, Kathmandu Medical College, Kathmandu, Nepal; 2 Department of Internal Medicine and Clinical Nutrition, Institute of Medicine, Sahlgrenska Academy at University of Gothenburg, Gothenburg, Sweden; 3 Patan Academy of Health Sciences, Lalitpur, Nepal; 4 Nordic School of Public Health NHV, Gothenburg, Sweden

**Keywords:** cardiovascular disease, health behavior, health promotion, Nepal, perceptions, qualitative research

## Abstract

**Background:**

Nepal currently faces an increasing burden of cardiovascular disease (CVD). Earlier studies on health literacy and the behavior dimension of cardiovascular health reported a substantial gap between knowledge and practice.

**Objective:**

This qualitative study aimed to deepen understanding of the community perspective on cardiovascular health from the patients’ viewpoint.

**Design:**

We conducted in-depth interviews (IDIs) with 13 individuals with confirmed heart disease, hypertension, or diabetes mellitus. All participants provided verbal consent. We used an IDI guide to ask respondents about their perception and experiences with CVD, particularly regarding causation and preventability. We manually applied qualitative content analysis to evaluate the data and grouped similar content into categories and subcategories.

**Results:**

Respondents perceived dietary factors, particularly consumption of salty, fatty, and oily food, as the main determinants of CVD. Similarly, our respondents unanimously linked smoking, alcohol intake, and high blood pressure with cardiac ailments but reported mixed opinion regarding the causal role of body weight and physical inactivity. Although depressed and stressed at the time of diagnosis, respondents learned to handle their situation better over time. Despite good family support for health care, the financial burden of disease was a major issue. All respondents understood the importance of lifestyle modification and relied upon health professionals for information and motivation. Respondents remarked that community awareness of CVD was inadequate and that medical doctors or trained local people should help increase awareness.

**Conclusions:**

This study provided insight into the perceptions of patients regarding CVD. Respondents embraced the importance of lifestyle modification only after receiving their diagnosis. Although better health care is important in terms of aiding patients to better understand and cope with their disease, interventions should be tailored to improve the community's cardiovascular health literacy and preventive practices.

Cardiovascular disease (CVD) causes one quarter of all deaths worldwide (1). Eighty percent of such deaths result from tobacco smoking, unhealthy diet, harmful use of alcohol, and physical inactivity (2). Because controlling negative behavior is crucial to curbing CVD and other non-communicable diseases (NCDs), current research has focused on behavior-related determinants (3).

Populated by 27 million people, Nepal is in epidemiological transition and battling a double burden of disease (4). Recent trends suggest that Nepal faces an increasing level of NCDs, including CVDs (5, 6). Population-based studies indicate a high burden of cardiovascular risk factors, particularly those related to behavior (4, 5, 7). In the absence of a national policy to steer programs for NCDs, current emphasis focuses more on the curative rather than preventive and promotive aspects. Unsurprisingly, cardiovascular health literacy in Nepal is poor (8–10).

To explore the health literacy and behavior dimension of cardiovascular health in Nepal, in 2010, we established the Jhaukhel-Duwakot health demographic surveillance site (JD-HDSS) in two urbanizing villages located near the capital city of Kathmandu (11). Among several population-based projects in JD-HDSS, the Heart-Health Associated Research and Dissemination in the Community has conducted studies on cardiovascular health including assessment of cardiovascular health literacy, measurement of the behavioral dimensions of cardiovascular health, and health promotional activities targeting young mothers.

Our recent quantitative assessment of general knowledge about cardiovascular health in JD-HDSS revealed that 44% of the population lacks sufficient understanding of the causes of heart disease and its prevention (10). Furthermore, a substantial gap exists between knowledge and practice regarding cardiovascular health.

Factors that affect cardiovascular health behavior can be understood through different lenses such as health behavior models (12), health locus of control theories (13, 14), and the concept of explanatory models (15). For example, social cognitive theory, a health behavior model developed by Bandura, proposes a tripartite reciprocal relationship between personal factors, environmental influences, and healthy behavior (16). Qualitative studies are particularly useful in understanding such relationships because they explore individuals’ views on cardiovascular health issues (17, 18). On the contrary, health locus of control describes individuals’ expectations regarding the effect of behavior on their health. Locus of control is essentially categorized as internal and external (13, 14). Explanatory models, which provide a framework to explain illness and treatment during the clinical process, seek to clarify five major dimensions of illness (etiology; time and mode of onset of symptoms; pathophysiology; course of sickness; and treatment) from the perspective of both patient and health care providers (15).

Ultimately, these theories and concepts aim to increase understanding of social behaviors underlying the clinical manifestations of illness and also help individuals recognize pathways that promote better health behavior. In this regard, qualitative data provide knowledge that helps achieve this aim. Furthermore, such data is useful for designing community-based cardiovascular interventions targeting either pre-pathogenesis or pathogenic stages of disease (19). The present qualitative study sought to deepen understanding of the community's perception of cardiovascular health from the patients’ viewpoint. We furthermore aimed to identify beliefs, perceived barriers, and level of awareness regarding CVD.

## Methods

### Setting and participants

The study was conducted between May and July 2013 in the JD-HDSS of Bhaktapur district in the Kathmandu Valley of Nepal (11). JD-HDSS has 2,712 households (1,557 in Duwakot and 1,155 in Jhaukhel) and a population totaling 7,612 and 6,057, respectively. Kathmandu Medical College (KMC) and Nepal Medical College operate community hospitals in Duwakot and Jhaukhel, respectively.

To obtain access to the knowledge sought in our study, we used the following inclusion criteria: patients with confirmed heart disease, hypertension, or diabetes mellitus for at least 1 year; stable condition at the time of interview; aged ≥20 years and of either sex; living in the JD-HDSS for at least 1 year prior to the study; and voluntary participation in the study.

Initially, participants were selected from the patient registry of the KMC Community Hospital. We recruited additional participants by asking initial respondents to identify other patients who would be willing to participate. Each participant was informed about study objectives 1 week before the interview. We scheduled appointments at participants’ convenience but within the regular working hours of the community hospital. Moreover, participants were reminded about the interview the evening before the appointment. No participants dropped out and none of the individuals we approached refused to participate in the study.

### Data collection

We conducted in-depth interviews (IDIs) with 13 patients to collect data, asking open-ended questions from an IDI guide that was developed using relevant literature (17, 20–28). Furthermore, we pre-tested the IDI guide with two patients, a 74-year-old male and a hypertensive 39-year-old female. After pre-testing, we made only minor changes (e.g. order of questions) to the initial guide. We included both pre-test interviews in our final analysis.

The first authors (NO, AV), who are PhD students at the University of Gothenburg, conducted the data collection process. NO gained previous experience in qualitative studies in Nepal while pursuing her Master in Public Health degree and AV previously conducted research in community-based cardiovascular health. Their PhD studies involve health literacy and the health promotion aspects of cardiovascular health. For this study, NO acted mainly as the facilitator and AV was the note taker. However, they participated equally in interactions with the respondents and during discussions of some topics that required additional explanation. Their participation allowed respondents to discuss relevant topics more freely and made the flow of interviews smoother.

During interviews, NO and AV asked participants about their perception of their general health; their knowledge and perception about CVD and its risk factors; and post-diagnosis lifestyle modifications; if any. Furthermore, they assessed the psychological and social impacts of CVD-participants’ feelings immediately after diagnosis and at present, and behavior of family members and other close relations. Finally, they asked respondents about the possibility of CVD prevention and sought their opinion regarding how prevention activities might be structured.

Each IDI began with broad questions (e.g. ‘What is health for you?’). Another question (i.e. ‘Can you tell us about how your disease started?’) helped respondents tell their stories in their own words and made the flow of the interview more comfortable for them. We used open-ended questions to elicit other responses. For example, in order to explore respondents’ experience of his or her condition, we asked: ‘What were your feelings and reactions when you first were diagnosed?’ and ‘How have your feelings regarding disease changed compared to ones how you felt at the beginning of your disease?’ Similarly, the questions related to lifestyle changes were: ‘Did you change your lifestyle after you had been diagnosed?’, ‘Are you facing any difficulty changing lifestyle?’, and ‘What do you think will make you change your lifestyle?’ Then, to explore perceptions regarding prevention of CVDs, we asked: ‘Do you think CVDs can be prevented?’ and ‘What can be done to raise awareness about heart disease in the community?’ Probing questions provided necessary clarification and additional opportunities for exploration.

NO and AV carefully noted all signs of respondents’ non-verbal communication, discussed the interviews among themselves, and included their observations in the results. Observations of emotional reactions complemented verbal information and deepened our understanding of perceptions and feelings.

All interviews were conducted in the Nepali language and each lasted about 1 hour. Both authors are fluent in Nepali language. Twelve interviews were conducted in a separate, well-lighted room in the Outpatient Department of KMC Community Hospital. We interviewed one respondent at her home because her age made travel difficult. Privacy was maintained during all interviews. Snacks (tea and biscuits) were available to all respondents during the interview and all received a travel allowance totaling Nepali Rupees 300 (US$3) at the end of the interview.

At the conclusion of each interview, NO and AV discussed the results among themselves, noting and emphasizing all important aspects. These discussions helped trace new information, detect repetition, and assess data saturation. Although they detected data saturation during the 10th interview, they conducted three more interviews to confirm that saturation had occurred.

### Data analysis

We used qualitative content analysis to manually evaluate all data (29, 30), focusing on manifest content (i.e. all visible and obvious components) (31, 32). An experienced professional translator transcribed all data accurately and verbatim from Nepalese to English. NO and AV examined the transcripts separately. They discussed the transcript until they reached a consensus on significant content areas, extracting meaning units and generating codes. Example of qualitative content analysis showing meaningful units, their condensation and abstraction is given in the Table 1. Next, NO and AV grouped similar content emerging from the analyses into various categories, subcategories, and sub subcategories which are presented in the Table 2.

**Table 1:** Example of qualitative content analysis showing meaningful units, their condensation and abstraction

Respondent	Meaningful units	Condensed meaningful units	Codes	Sub subcategories	Subcategories	Categories
R7	Causes of heart disease are smoking … and excessive alcohol …	Smoking and excessive alcohol cause heart disease	Smoking Alcohol excess	Smoking Alcohol	Risk factors	Heart disease linked to diet and other health behaviors
					
R13	Festivals have effect on health and heart as there is more consumption of alcohol and high content fats, as well as spicy, oily foods during festivals.	Festivals affect the heart due to high consumption of alcohol, spices and fat.	Festivals	Effect of tradition and culture	Sociodemographic environment		

**Table 2:** Qualitative content analysis: categories, subcategories and sub subcategories

Categories	Subcategories (sub subcategories)
Heart disease linked to diet and other health behaviors	General health (understanding, responsibility, health problems in the community)
	Heart disease
	Risk factors (diet, physical activity, smoking, alcohol, body weight blood pressure, others)
	Sociodemographic environment (effect of tradition and culture, role of peers)
Personal distress, financial difficulties and family support	Personal (feelings at diagnosis and at present, support of family and neighbors)
	Health care
	Financial impact
Lifestyle modifications are well understood, but difficult to follow	Efforts
	Continuity and success
Awareness of heart disease is too little, too late	Level of awareness in the community suggestions for improving awareness

### Ethical considerations

The Institutional Review Board at KMC gave ethical approval for this study. We explained the study objectives and the risks and benefits of participation to all respondents before beginning the interviews. All respondents provided verbal consent. Moreover, we obtained additional consent before taking notes and tape-recording the interviews. During sensitive questions, we considered respondents’ emotional status as cues regarding whether to probe further on that particular issue. Finally, we addressed any health-related queries raised by the respondents.

We assured all respondents regarding the confidentiality of their information. There were no external observers during the interviewing process, and all documents, including notes and tape recordings, were securely stored and accessible only to the research team.

## Results

We interviewed 13 patients with established cardiometabolic disease. The average age of patients was 59.5±13.8 years (64.8±10.6 years for males and 54.9±15.2 years for females). Characteristics of the participants are presented in Table 3.

**Table 3:** Demographic characteristics of the participants

Respondent	Age (years)	Sex	Ethnicity	Education	Occupation	Disease
R1	74	Male	Newar	No formal education	Retired farmer	Hypertension
R2	39	Female	Brahmin	No formal education	Housewife	Hypertension
R3	50	Male	Brahmin	Masters level	Teacher	Valve replacement
R4	58	Male	Newar	Grade 10	Retired government employee	Ischemic heart disease
R5	40	Female	Brahmin	Grade 4	Housewife	Hypertension
R6	70	Female	Newar	No formal education	Housewife	Hypertension
R7	79	Male	Newar	Grade 8	Farmer and traditional healer	Hypertension
R8	45	Female	Newar	No formal education	Housewife	Hypertension and diabetes
R9	62	Male	Chhetri	Grade 10	Ex-army	Hypertension and diabetes
R10	66	Male	Chhetri	Grade 10	Ex-army and homeopathy	Ischemic heart disease
R11	79	Female	Chhetri	No formal education	Housewife	Ischemic heart disease
R12	57	Female	Kirat	No formal education	Owns a small eating outlet	Arrhythmia
R13	54	Female	Chhetri	No formal education	Farming	Ischemic heart disease

### Heart disease linked to diet and other health behaviors

#### General health

All respondents viewed health as precious and they connected ‘being healthy’ with the ability to work without difficulty. They assumed responsibility for their own health and believed that neglecting one's own health resulted in disease. Conversely, some respondents believed that good health was a family responsibility and another believed that health was determined by God.

The respondents considered high blood pressure (‘BP’ or ‘pressure’) and diabetes mellitus (‘sugar’) as the most prevalent health issues in their community. They considered respiratory problems (asthma, chronic obstructive pulmonary disease, tuberculosis, and acute respiratory infections) as common ailments. Respondents presumed that alimentary conditions such as gastritis (‘gastric’) and vomiting were common. Respondents also recalled seeing a patient with stroke (‘paralysis’).

Respondents’ opinion was divided regarding the community burden of CVD: some thought it was common and others had not ‘*found any heart patient in the community*’. Respondents linked most ailments, particularly diabetes and CVD, to diet (dietary changes or consuming unhygienic, contaminated, or adulterated food). They also mentioned environmental factors (pesticides and air pollution caused by the nearby brick factories).

#### Heart diseases

Most respondents appeared confident when answering the IDI questions. CVD (‘*mutukorog*’ in Nepali) had different meanings. Some respondents described it as ‘pain in the heart’ or ‘fast heart beating’, but most connected CVD with specific causes (smoking, hereditary, ignorance, negligence, age, blows to the heart while fighting, etc.) or physical conditions (a hole in the heart, etc.). Others associated CVD with psychological problems such as fear of gatherings or being afraid when others are speaking.

#### Risk factors

All respondents understood the negative impact of behavioral risk factors on CVD, including unhealthy diet, physical inactivity, smoking, and excessive alcohol consumption. However, a majority of them received such information from doctors only after being diagnosed with CVD.

… from doctors … I was more aware about heart disease after my diagnosis. Previously I knew about heart disease but … I thought … I won't get it so why worry …. (R10)

Some of the respondents said they learned about cardiovascular health mostly from newspapers and television programs.

##### Dietary habits

Respondents considered diet a major contributor to CVD. Most identified consumption of salty, fatty, and oily food as a frequent cause of cardiac ailments and a few regarded spicy food and meat as causes.

… I think excessive fatty meat (‘boso’) … and oil content … and fatty diet (‘Chillo khana’) … is harmful for heart and health. (R12)

… oily and spicy food (‘Chillo piro khana’) … they increase blood pressure …. (R5)

Some respondents considered stale food an important reason for CVD.

In my view … if fresh and proper diet is consumed then there is no heart problem … it has rather good effect. (R12)

Respondents also associated CVD with the decreased availability and consumption of food that commonly occur with poverty. Other perceived dietary links included adulterated food, a rich diet, or adopting a new eating pattern. Respondents also connected CVD with food temperature, particularly for fruit. Such perception can be influenced by cultural beliefs.

Fruits which are cold also cause problem in the heart. (R12)

##### Physical activity

A majority of respondents associated higher risk for CVD with lack of physical activity, believing that such activity makes people healthy and keeps heart disease away by ‘maintaining pressure of our body’ and by ‘protection of the heart’. On the contrary, respondents linked sedentary lifestyle to disease pathology.

… By doing work (‘Kaam garera’), we will not suffer from any diseases … those who sit without doing any work … they suffer from disease. (R11)

A majority of respondents thought that walking for 1 hour each morning was good for the heart. They also thought it even more important that heart patients maintain regular physical activity in the form of morning walks and other physical exercises.

… Walking around (‘heend-dool’) has good effect on health … on heart … when we walk daily, it maintains the pressure of our body … and we will be free from disease. (R6)

However, some respondents believed that walking or physical exercise had no effect on the heart.

I don't think walking affects the heart. (R7)

##### Smoking

All respondents believed that smoking causes CVD, and many thought that the effect of smoking on the heart is direct.

Cigarette smoking has bad effect on health … as harmful smoke (‘dhuwan’) enters inside us … and can create high pressure and heart problem. (R6)

Many respondents thought that the lungs mediate the effect of smoking on the heart.

Smoking causes difficulty in the lung … and due to trouble in the lungs, it affects the heart. (R1)

Many respondents had difficulty or expressed uncertainty while answering questions regarding the impact of smoking on health and the heart, probably due to a lack of knowledge and understanding.

###### Alcohol consumption

Most respondents thought that alcohol consumption was harmful to the heart, and many assumed that alcohol ‘contained unnecessary adhesives and the contaminated blood subsequently damaged the heart’.

There is a bad effect of alcohol on heart and health … one of my relatives drank too much and his heart burst (‘Mutu futyo’) … and he bled from his mouth … and he died. (R13)

On the contrary, some respondents believed that small amounts of alcohol are good for the heart.

[Alcohol] may even act like a medicine during cold, fever … it is when people drink a lot, it causes problems …. (R12)

##### Body weight

Many respondents linked overweight (‘*Mottaunu*’) with heart disease. Some of them thought that increased weight causes heart disease due to fat deposition in the body or because a fat person eats more but does less work.

Too much weight makes pressure on our body … makes us feel uneasy … causes difficulty in breathing while walking and affects heart. (R2)

On the contrary, some respondents shared that anyone can develop heart disease irrespective of their body weight.

… anyone, whether thin or fat … can have heart disease. (R9)

##### Blood pressure

A majority of respondents were aware that high blood pressure has negative effects on the heart. ‘Increased blood pressure affects our heart functioning … it causes irregular pumping of blood from our heart’ (R8).

Some respondents also believed that there is connection between a person's weight and blood pressure and that excess weight causes high blood pressure.

When asked if there are other risk factors that cause heart disease, respondents did not add other causative factors.

#### Sociodemographic environment

##### Effect of tradition and culture

Nepal's rich culture and traditions involve different religious rituals, usually accompanied by the intake of high-calorie food. A majority of respondents described a relationship between cultural practices and heart disease.

During festivals, people take more oily and spicy food than usual … especially some ethnic groups … they take alcohol during festivals. It can create problem for the heart. (R2)

… tension and load even during preparation and … conducting cultural ceremonies can be harmful for the heart. (R4)

##### Role of peers

Many respondents emphasized that peer pressure plays an important role in unhealthy lifestyles (smoking or alcohol consumption) and the development of CVD.

I got heart disease due to the influence of my friends. (R10)

Peer influence (‘sangat’) makes it difficult to change behavior. (R5)

##### Effect of socioeconomic status

A majority of respondents offered strong opinions regarding the association between financial status and CVD. Some thought that wealthy people were more susceptible to heart disease due to sedentary lifestyle, overeating, or fear of losing their property. Others thought that poor people were more susceptible to CVD due to the stress of earning money, poor diet, or working more.

### Personal distress, financial difficulties and family support

#### Personal experiences

##### Feelings at diagnosis and at present

We asked respondents to describe their feelings at diagnosis and how they perceive their condition now. Respondents reported depression and stress at the time of diagnosis due to inevitable changes, including lifestyle modification, life-long medication regimens, and physical restrictions resulting from shortness of breath and fatigue. They recalled fearing the possibility of surgery or immediate death. Moreover, many respondents looked worried about limited capacity or inability to work due to disease, particularly if their main occupation was farming.

Over time, a majority of respondents started to consider their condition as normal because they thought that it was possible to manage their disease with medication and lifestyle modification.

I didn't go for treatment because I was afraid of operation. My children were still small … I thought … if I die … what would happen to them? After been diagnosed my first feeling was fear of not getting cured … Nowadays I am taking my disease much easily because I have less pain and trouble due to medicines. (R4)

However, some respondents remained as tense and stressed about their disease as they were at the time of diagnosis. Some of them reported consistent fear of large gatherings and loud discussions. Others described a fear of falling while walking alone.

##### Support of family and neighbors

All respondents reported receiving excellent family support following diagnosis. Family reactions included worrying about the patient's health, supporting lifestyle modifications, protecting the patient from stress and negative information from outsiders, and providing financial support. Some respondents said their families were stressed after learning about the diagnosis because it would be difficult to pay for treatment.

When my family came to know I had a heart problem they were tense. It was very hard for them to manage money for my treatment, they were also about to sell land for my treatment. (R13)

When my family came to know about my disease they became stressed because they had to cook separately for me from now on. (R9)

Some respondents reported family stress regarding their disease because family members did not understand the cause and consequences of CVD. On the other hand, neighbors and friends encouraged them to change their lifestyle and have regular checkups.

There was no change in behavior of the people. Everyone treated me well and also asked me regarding prevention of heart disease. (R13)

#### Experience of health care

Although most respondents were satisfied with the treatment prescribed by their health care providers, some complained about lack of information and clarity regarding disease.

I am not much satisfied with the treatment provided by the doctor. He did not clarify to me about the disease from which I am suffering. (R9)

#### Financial impact

Most respondents experienced economic difficulties in managing their disease.

… It is difficult for me … to afford treatment … it is due to my low income. (R5)

Many respondents depended on others to manage funds for their treatment and medicine.

… difficult to manage money … but daughters and son are helping [me] financially. (R4). Only a few respondents had the financial resources to easily pay for treatment.

… With my husband's pension money, it was easy to buy my medicines …. (R11)

### Lifestyle modifications are well understood, but difficult to follow

#### Efforts at lifestyle modifications

All respondents understood that lifestyle modification could decrease the impact of their disease and all said they were making changes in their lifestyle (reducing salt intake or eating less oily food and meat). Other lifestyle modifications included exercise (‘*heed dooly garner*’), stress reduction (‘*tension kami garner*’), and reduced smoking and alcohol intake.

Although health concerns limited physical activity for some respondents, many said they were exercising regularly. Some respondents did not believe that physical activity has a positive impact on cardiovascular health. They stressed that their doctor had not advised any increase in physical activity.

#### Continuity and success

Many respondents reported difficulty in changing their diet according to the doctor's advice. Some male respondents finally quit smoking only after several years of unsuccessful attempts. ‘Being a diseased person … there are several hindrances …. In gatherings, I can't eat as I like due to my disease [giggles]’ (R2).

Another female respondent said that she was a vegetarian and had already consumed less salty and oily foods even before her diagnosis.

### Awareness of heart disease is too little, too late

#### Level of awareness in the community

All respondents acknowledged that community awareness of CVDs was not adequate.

People here generally are not aware of heart diseases … how they occur … they don't care much [‘Matlab chhaina’]. (R3)

Respondents opined that most people do not think about their health until disease had already occurred.

Only those who have suffered … talk about heart disease … the rest of the people are less concerned about it. (R5)

#### Suggestions for improving awareness

When we asked respondents about the possibility of CVD prevention in their community and invited their suggestions regarding implementation, they emphasized dietary aspects (low salt and low fat intake), stress avoidance, abstaining from smoking and alcohol consumption, and physical activity through morning walks and yoga. However, several respondents commented about the difficulty of sustaining lifestyle modification over time.

Most respondents recognized the need for community awareness programs regarding cardiovascular health and suggested that medical doctors or other trained local people should educate the public. Respondents believed that this approach would help people improve their own health and encourage them to seek necessary medical care.

Doctors should inform [the local people] about how to maintain health … awareness should be raised through demonstration … negative and positive consequences should be informed to the people … awareness should be created among the people … for example, to the children in school … to the elderly in a place where they can gather. … to the parents of the children …. (R9)

## Discussion

This study is the first in Nepal to highlight the perceptions of people living with cardiometabolic disease regarding risk factors and the dynamics of their lifestyles, experiences, and perspectives of CVD prevention. Our qualitative study design was useful for exploring perceptions at the community level and also provided added potential for use as a development tool during future health education interventions.

The majority of our respondents perceived hypertension and diabetes as common health problems in their community, confirming findings reported in other settings (20, 33, 34). Moreover, a majority of respondents understood that risk factors (unhealthy diet, low physical activity, smoking, alcohol consumption, and stress) affect the development of CVD. Some respondents offered strong opinions about the relation between financial status and heart disease, believing that prosperity caused heart disease through fear of losing property, sedentary lifestyles, and poor dietary habits. No one mentioned the impact of heredity. Similar findings have been described elsewhere as well (35).

Because our study respondents live in a peri-urban community, they had adopted unhealthy lifestyles that predisposed them to developing cardiometabolic diseases. Their misconceptions about healthy diet raised particular concern. For example, we observed inadequate knowledge and understanding about the importance of regularly consuming fruits and vegetables. In addition, respondents viewed the typical and traditional South Asian diet, which is rich in ‘ghee’ (clarified butter), oil, and fried foods, as normal. Along with sedentary lifestyle, dietary misconceptions increase the risk of developing CVD among the general Nepalese population (9).

Most of our respondents had not considered themselves at risk of CVD and only learned about cardiovascular health from doctors post-diagnosis. Newspapers and television programs provided some health-related information but unlike the migrant population of Delhi (34), our respondents paid little heed to such reports. In Nepal, people view medical personnel as the main source of information about health issues but such professionals may lack adequate training to counsel patients about lifestyle modifications and other aspects of patient care. Coupled with a lack of structured cardiovascular health education programs, this can negatively affect the quality of counseling provided to the patients (21).

It is useful to discuss our respondents’ perspectives in the context of health locus of control (13, 14). A majority of respondents did not emphasize the importance of adopting healthy lifestyles before they were diagnosed with CVD. This hints at ‘externality’ of the locus. Our recent quantitative study in JD-HDSS confirms a strong conviction that a higher power determines health (‘chance externality’) (10). Similarly, a Newar community on the outskirts of Kathmandu Valley strongly believes in supernatural causes of illness (36), and South Asian immigrants in the United Kingdom attribute illness to God's will (37). However, after diagnosis most of our respondents believed that their condition resulted from their own unhealthy behavior (‘internality’). Furthermore, in matters regarding health-related information and disease management, the health locus of control was on doctors or medical professionals (‘powerful others externality’). In other words, after respondents became ill, they expected doctors to provide health-related information and care. For example, respondents who did not receive counseling for physical activity concluded that physical activity was not important. Combined with a lack of internal motivation, this dependence on health personnel shows that respondents have an external locus of control (38). Studies in other low-income countries (39) and also among South Asian immigrants in the United Kingdom (37) have reported a similar health locus into ‘powerful others’. Furthermore, apart from the health locus of control perspective, beliefs regarding heart disease and experiences acquired through interactions with health care providers, other patients, and family also affect behavioral changes (40). Among these, the patient-provider interface is best explained through the concept of the explanatory model, which facilitates communication between patients and health care providers to improve patient understanding and identify areas of conflict (15). Indeed, researchers and health planners must remember these aspects when contemplating health promotional activities for cardiovascular health for both patients and the general population in this urbanizing Nepalese community (41).

Despite diverse demographic characteristics, geographical and cultural background, and types of health care facilities available, people with CVD worldwide share similar emotional, psychological, and social experiences (42). In this regard, our respondents also experienced depression, frustration, and fear of dying when initially diagnosed with disease. Many respondents experienced additional stress from having to change their lifestyle according to the doctor's advice. However, respondents gradually developed different coping strategies to alleviate the impact of disease on their lives. Perhaps the most important coping mechanism was the involvement of family and friends while dealing with the disease. Such interactions provided crucial social, psychological, and emotional support and encouragement, a phenomenon shared by cardiac patients in other settings (43–45). Another coping strategy involved making necessary adjustments to new circumstances. Despite the difficulties, all respondents talked about the changes they made in their diet (decreased consumption of salt, spices, and red meat; smoking cessation; and reduced alcohol consumption). Several respondents experienced physical limitations such as shortness of breath and fatigue but accepted their new life situation and made behavior changes accordingly. For example, they reduced daily activities (working at the farm or doing household chores) or performed them at a slower speed. Due to physical discomfort such as headache and irritation, several females avoided large gatherings, preferring to spend more time in a calmer home environment. Nevertheless, the severity of the disease influences adaptation, a family's ability to manage finances for treatment, the quality of family and social support, and individual understanding of the disease (42). Similar to patients in other settings (33, 43), our respondents viewed CVD as an illness that can be controlled with medication and lifestyle modification. In addition, they faced similar challenges in sustaining lifestyle modification (34, 37, 43). However, patients from high-income countries are more skeptical about the importance of lifestyle modification (33).

As stated earlier, our respondents attempted lifestyle and behavior modification only after receiving a diagnosis. As explained by the health belief model, few individuals take preventative action until they 1) perceive themselves susceptible, and 2) believe that the disease may potentially present serious consequences (16, 46). Perceived benefits of engaging in a particular health-related behavior and perceived barriers toward adopting such behavior are also important components of the health belief model, which explains why our respondents changed their lifestyle only after diagnosis. Because CVD is usually considered a disease of aging, most young and middle-aged adults underestimate their own susceptibility. Hence, initiating the process of lifestyle modification usually requires a trigger, which can be internal (e.g. disease), external (e.g. health promotion programs), or both (35). On the other hand, the theory of reasoned action and the theory of planned behavior propose three major components that influence behavioral intention: attitude toward behavior, perceived behavioral control, and subjective norms (12, 46). In our context, the role of the subjective norm component of the theory for developing behavioral intention toward cardiovascular health is particularly important. Subjective norms influence not only a person's beliefs about what others think he or she should do (normative beliefs) but also the motivation to behave according to others’ wishes. The degree of social influence depends upon the importance an individual attaches to societal opinion (46). For example, Nepalese society views overweight and a big belly as signs of positive health and prosperity (47). Also, the Nepalese people view food cooked with less salt and oil or not deep-fried as unappetizing or inferior. Many of our respondents attached little importance to fruit, thinking it causes coughs, colds, and CVD: ‘*Fruits which are cold also cause problem in the heart*.’ There is little doubt that such perceptions play crucial roles in establishing people's behavior (48).

In summary, the present study increases understanding of perceptions about cardiovascular issues in a peri-urban community, particularly from the patient's perspective. However, we must consider some possible limitations. Our sample size was small and our CVD patients were heterogeneous in terms of the type, severity, and duration of disease. We used non-probability sampling to select respondents, thus limiting the transferability of our findings. Moreover, there is a possibility of recall bias regarding feelings and emotions experienced immediately after confirmation of disease if the diagnosis was not recent.

## Conclusions

This qualitative study, conducted in a peri-urban community in Nepal, explored the perceptions and experiences of CVD patients regarding their illness, the psychological and social impacts of disease, and adaptive strategies. Our findings suggest the need to develop and implement different health education programs to address the lack of awareness and existing misconceptions regarding the importance of cardiovascular health. Exploring perceived susceptibility toward cardiometabolic diseases, understanding the perceived barriers and potential benefits of behavior modification, and assessing preparedness for interventions will require subsequent study among different sociodemographic groups within the general population.
